# Establishment of an HSV-1 Mouse Model with Cutaneous Lesions

**DOI:** 10.3390/pathogens15080787

**Published:** 2026-07-24

**Authors:** Hye-Myung Ryu, Bushra Riaz, Seonghyang Sohn

**Affiliations:** 1 Department of Microbiology, Ajou University School of Medicine, Suwon 16499, Republic of Korea; naya101@hanmail.net; 2 Department of Biomedical Sciences, Ajou University School of Medicine, Suwon 16499, Republic of Korea; bushra0869@ajou.ac.kr; 3 BK21 R&E Initiative for Advanced Precision Medicine, Graduate School of Ajou University, Suwon 16499, Republic of Korea

**Keywords:** Herpes simplex virus type 1 (HSV-1), mouse model, cutaneous HSV infection, antiviral therapy

## Abstract

Herpes simplex virus type 1 (HSV-1) causes recurrent mucocutaneous lesions, yet existing animal models incompletely recapitulate characteristic skin manifestations. Here, we established a mouse model of cutaneous HSV-1 infection by combining epithelial barrier disruption with localized viral inoculation. Superficial scarification of the ear followed by HSV-1 exposure resulted in consistent lesion formation. Administration of hydrocortisone further increased the incidence of lesions, highlighting the critical roles of epithelial integrity and host immune regulation in the pathogenesis of HSV-1 infection. Using this model, we evaluated the antiviral efficacy of Acyclovir. Treatment significantly reduced lesion severity, lesion size, and viral gene expression, indicating partial suppression of viral replication. Notably, acyclovir treatment was associated with increased expression of T-bet and Foxp3 in lymphoid tissues, suggesting modulation of both effector and regulatory immune responses. Collectively, this model successfully reproduces localized HSV-1 skin lesions, serving as a useful platform for investigating viral pathogenesis and evaluating antiviral therapies. However, further studies, including direct viral quantification and histopathological analysis, are required.

## 1. Introduction

Herpes simplex virus infection is a highly prevalent human viral disease caused by herpes simplex virus type 1 (HSV-1) and type 2 (HSV-2). It is characterized by recurrent mucocutaneous lesions and the establishment of lifelong latency in sensory neurons [1,2]. Among its clinical manifestations, vesicular and ulcerative skin lesions are a hallmark feature that reflects both viral replication and host immune responses [3]. These lesions are responsible for significant morbidity and serve as important endpoints for evaluating antiviral efficacy [4].

Mouse models have been extensively used to investigate HSV pathogenesis and to evaluate antiviral compounds due to their experimental accessibility and well-characterized immune system [5,6]. However, most conventional murine models do not reliably reproduce the characteristic skin lesions observed in human HSV infection. In many cases, infection results in subclinical viral replication or rapid systemic spread without the development of localized vesicular lesions [7,8]. This limitation has reduced the translational value of existing models, particularly for studies assessing therapeutic efficacy.

Acyclovir is the standard antiviral treatment for HSV infections. As a guanosine analog, it is phosphorylated by viral thymidine kinase and subsequently inhibits viral DNA polymerase, thereby suppressing viral replication [9,10,11]. Although acyclovir effectively reduces lesion severity and viral shedding, it does not eradicate latent infection or completely prevent recurrence. Accordingly, reliable animal models are essential for evaluating antiviral efficacy and for developing improved therapeutic strategies against HSV-1 infection.

To overcome the limitations of conventional models, several approaches been explored, including modification of inoculation routes [12], use of high viral titers [13], and use of immunocompromised [14] or genetically modified mice [15]. Although these approaches have partially improved lesion development, they often show substantial variability, limited physiological relevance, or reduced applicability to drug development studies. Thus, there remains an unmet need for a reproducible mouse model that consistently develops HSV-specific skin lesions under controlled conditions.

In this study, we aimed to establish a mouse model of localized cutaneous HSV-1 infection that consistently produces skin lesions. By optimizing both infection parameters and host conditions, we developed a practical platform for investigating HSV pathogenesis, host immune responses, and the therapeutic efficacy of antiviral agents.

## 2. Materials and Methods

### 2.1. Animals and Housing Conditions

Five-week-old male ICR mice were used in all experiments and maintained under specific-pathogen-free (SPF) conditions. The mice were divided into three groups: an uninfected control group (*n* = 20), an HSV-only group (*n* = 20), and an HSV plus hydrocortisone group (*n* = 80). Food and water were provided freely. All animal experiment procedures were strictly reviewed and approved in accordance with the guidelines established by the Ajou University Animal Care and Use Committee (IACUC-2018-0017). Sacrifices for additional experiments were performed using a CO_2_ chamber (set to 10–20% of chamber volume per minute) or cervical dislocation.

### 2.2. HSV-1 Inoculation Procedure

To establish the infection model, the epithelial barrier on the inner surface of the mouse ear auricle was superficially disrupted by gentle needle scarification, and 20 μL of HSV-1 (F strain; 1 × 10^6^ PFU/mL, was donated by the National Institute of Health of Korea in 1993), amplified in Vero cells, was applied to the scarified area. All viral experiments were performed in a Biosafety Level 2 (BSL-2) laboratory (LML08-1131), and the dose was selected based on a previously established protocol [16]. Mice were inoculated twice at 10-day intervals to provide repeated viral exposure. Beginning one day after each inoculation, hydrocortisone (150 μg per mouse) was administered intraperitoneally once daily for 5 consecutive days. The administration of hydrocortisone was to promote viral replication by suppressing the immune response [17]. When HSV was co-administered with hydrocortisone in mice, cutaneous symptoms developed in 15% of the subjects, compared to 5% in the group administered with HSV alone. These symptoms manifested 7 days post-viral inoculation. Starting from the onset of symptoms, acyclovir treatment was initiated or further experiments were conducted. Clinical signs were monitored on a daily basis throughout the experimental period. Furthermore, to minimize observer bias, clinical lesion scoring was performed independently by two investigators who were blinded to the treatment groups.

### 2.3. Pharmacological Treatment

To evaluate the therapeutic efficacy of acyclovir, acyclovir cream from GC Biopharma (Yongin, Republic of Korea) was used. Mice received topical application of acyclovir ointment once daily for 5 consecutive days, starting at the onset of HSV-1 symptoms, at a dose of 1 mg per mouse. Animals were observed daily, with particular attention to the appearance, location, and progression of skin lesions. For all experimental evaluations, symptomatic animals were randomly assigned to each treatment group (*n* = 3 per group).

### 2.4. RNA Extraction and cDNA Synthesis

Total RNA was isolated from the spleen and lymph node tissues using TRIzol reagent (Thermo Fisher Scientific, Waltham, MA, USA) according to the manufacturer’s protocol [18]. The concentration and purity of the extracted RNA were determined using a NanoDrop spectrophotometer before reverse transcription. For cDNA synthesis, 1 μg of total RNA was converted into first-strand cDNA using the PrimeScript™ cDNA Synthesis Kit (Takara Bio Inc., Shiga, Japan) with DNase I (Thermo Fisher Scientific) following the manufacturer’s instructions.

### 2.5. Quantitative Real-Time PCR Analysis

Gene expression was quantified by real-time PCR using SYBR Green PCR Master Mix (Applied Biosystems, Foster City, CA, USA) on a 7500 Real-Time PCR System (Applied Biosystems). Each reaction was performed in a total volume of 20 μL containing 1 μL of cDNA template, and all samples were analyzed in duplicate. The amplification protocol consisted of an initial denaturation step at 94 °C for 2 min, followed by 40 cycles of denaturation at 94 °C for 3 s, annealing at 55 °C for 30 s, and extension at 72 °C for 30 s, with a final extension step at 72 °C for 10 min.

The expression levels of T-bet, Foxp3, and HSV-1 ribonucleotide reductase genes (UL39 and UL40) were determined by qRT-PCR. Relative transcript abundance was calculated using the 2^−ΔΔCt^ method. Expression levels were normalized to the housekeeping gene β-actin and expressed as fold changes relative to the control group. The primer sequences used in this study are listed in Table 1.

### 2.6. Statistical Analysis

Statistical comparisons between experimental groups were performed using an unpaired Student’s *t*-test. Data are presented as mean ± standard deviation (SD). Differences were considered statistically significant when *p* < 0.05. All statistical analyses were carried out with GraphPad Prism (version 8.3.1) for Windows (GraphPad Software, La Jolla, CA, USA).

## 3. Results

### 3.1. Establishment of an HSV-1 Infection Model

**Figure 1:**
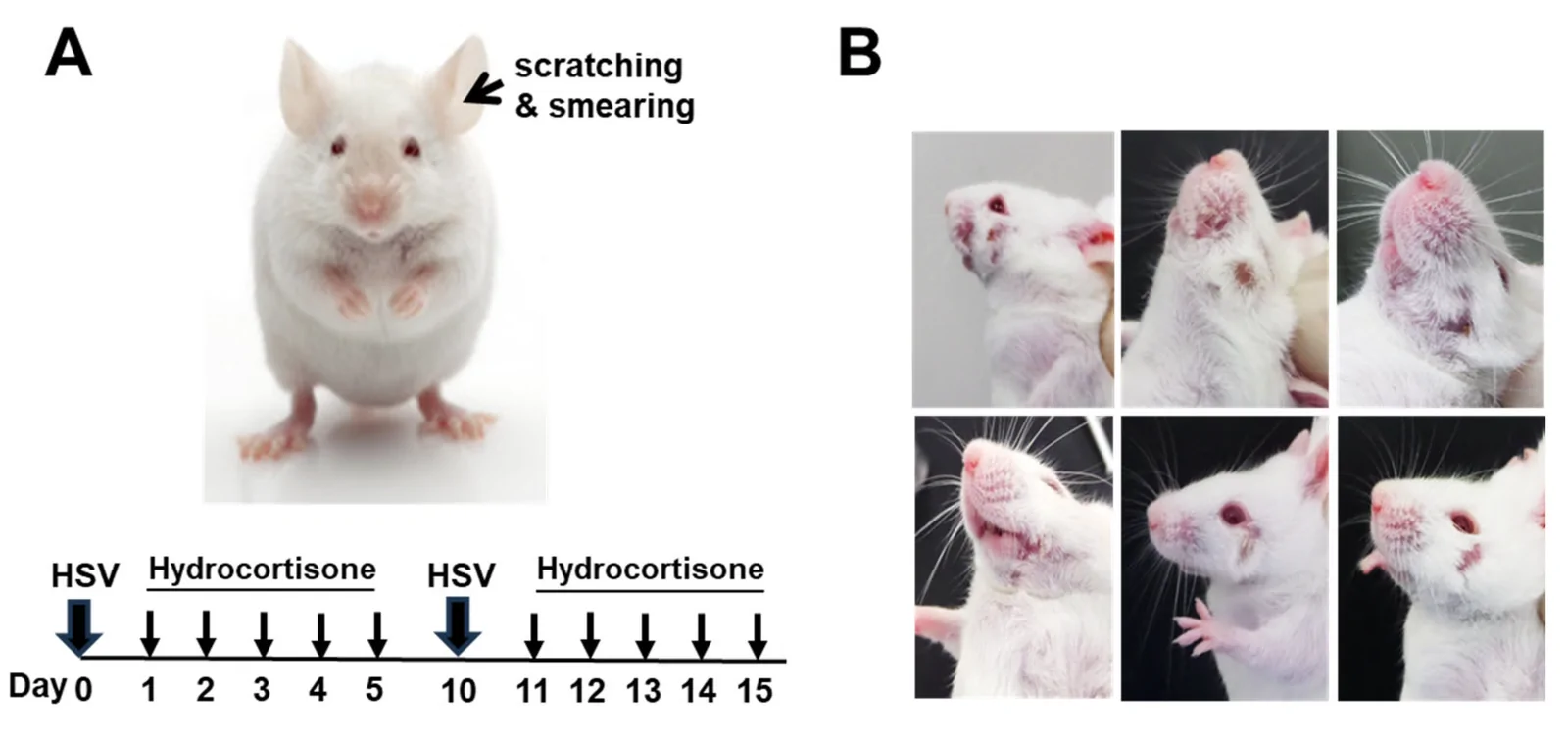
Establishment of a cutaneous HSV-1 infection model. (**A**) HSV-1 was inoculated onto the inner surface of the ear auricle after superficial scarification, resulting in reproducible skin lesion formation. Hydrocortisone was administered after infection to promote lesion development. (**B**) Following HSV-1 inoculation and subsequent hydrocortisone treatment, mice developed localized facial skin lesions without any evidence of systemic symptoms.

To establish an HSV-1 infection model, mice were inoculated with the virus via several routes, including oral, ocular, paw pad, and intraperitoneal administration; however, none of these approaches produced detectable clinical symptoms. In contrast, when HSV-1 was applied to the inner surface of the ear auricle following superficial needle scarification, reproducible cutaneous lesions developed (Figure 1A). These findings indicate that disruption of the epithelial barrier is essential for the successful induction of localized cutaneous HSV-1 infection in this model. In addition, hydrocortisone-mediated suppression of the host immune response enhanced lesion development, resulting in a more consistent and robust disease phenotype.

### 3.2. Analysis of Clinical Characteristics

Following HSV-1 inoculation and subsequent hydrocortisone treatment, mice developed localized facial skin lesions without any evidence of systemic symptoms. The mean area of the lesions was 6.5 ± 3.4 mm^2^ (Figure 1B). This restricted distribution of lesion indicates that the model primarily reflects localized cutaneous HSV-1 infection rather than systemic disease.

### 3.3. Acyclovir Inhibits Viral Replication and Improves Clinical Symptoms

**Figure 2:**
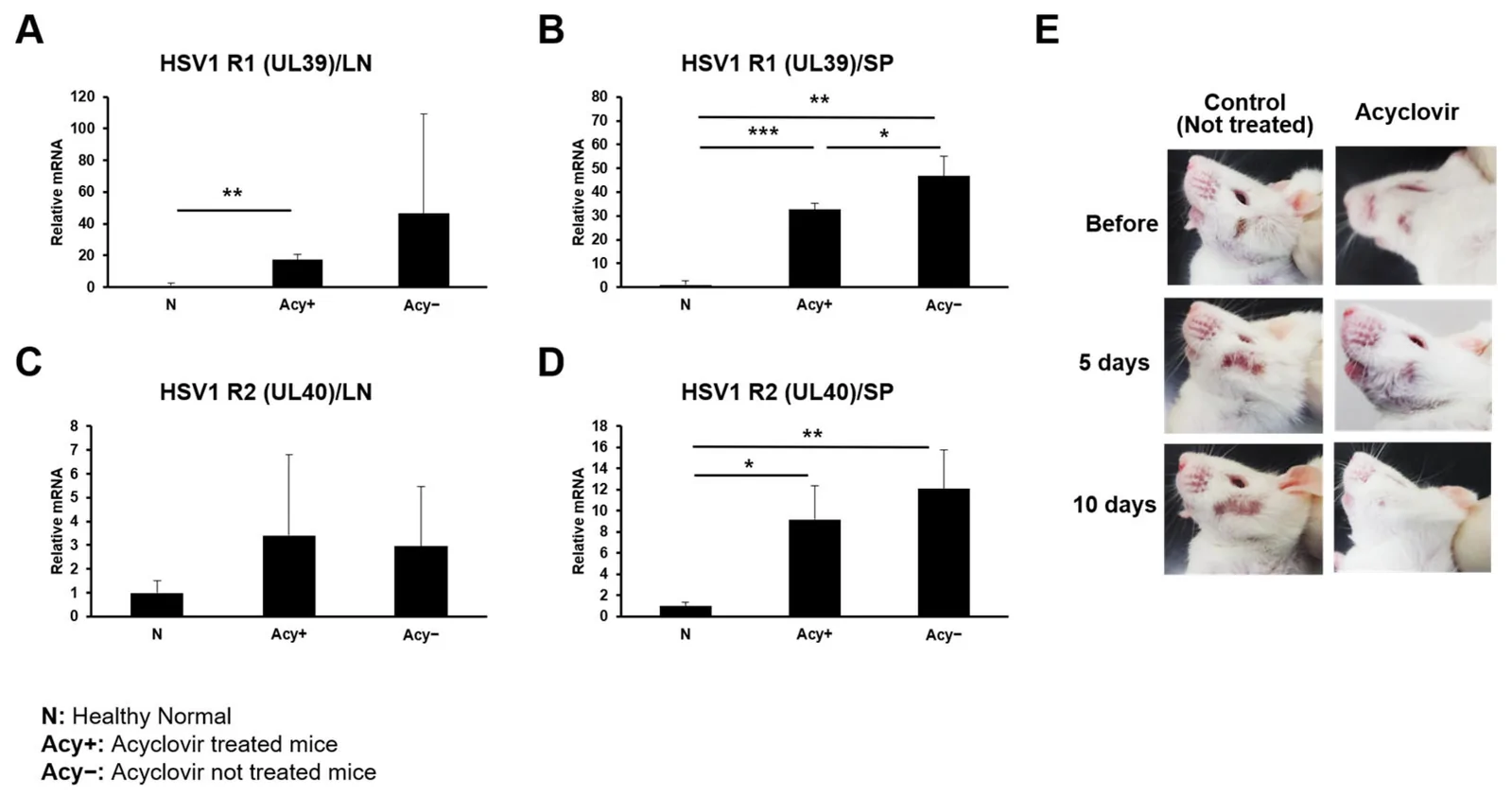
Effect of acyclovir on HSV-1 viral gene expression and skin lesions. (**A**,**C**) Expression of the HSV ribonucleotide reductase subunits R1 (UL39) and R2 (UL40) in lymph nodes was analyzed by real-time RT-PCR. HSV-1 infection increased the expression of both R1 and R2, with R1 showing a significant increase compared with the normal control group. (**B**,**D**) In the spleen, HSV-1 infection significantly upregulated the expression of both R1 and R2. Treatment with acyclovir reduced R1 expression and produced a decreasing trend in R2 expression, although both remained elevated relative to the normal control group. Data are presented as the mean ± SEM. Statistical significance is indicated as follows: (* *p* < 0.05, ** *p* < 0.01, *** *p* < 0.001). (**E**) Representative images of skin lesions before and after acyclovir treatment.

To assess viral replication, the expression levels of HSV ribonucleotide reductase subunits R1 (UL39) and R2 (UL40) were quantified in lymph node and spleen tissues by real-time RT-PCR. In lymph nodes, HSV-1 infection increased the expression of both R1 and R2. Although these increases were not statistically significant overall, R1 expression was significantly higher than that in the uninfected control group (*p* < 0.05) (Figure 2A,C). In the spleen, HSV-1 infection significantly upregulated the expression of both R1 and R2 compared with controls. Treatment with acyclovir reduced R1 expression relative to untreated infected mice, although expression levels remained higher than those in the normal control group (Figure 2B). Similarly, R2 expression showed a decreasing trend following acyclovir treatment, but the reduction did not reach statistical significance and remained above baseline levels (Figure 2D). Collectively, these results indicate that acyclovir partially suppresses viral ribonucleotide reductase expression, consistent with inhibition of HSV-1 replication in this model. Furthermore, acyclovir treatment significantly decreased the lesion area from 5.85 ± 3.0 mm^2^ before treatment to 0.46 ± 0.2 mm^2^ at day 10 (*p* < 0.01), accompanied by a marked improvement in the severity of skin lesions (Figure 2E).

### 3.4. Effects of Acyclovir on Immune-Related Gene Expression

**Figure 3:**
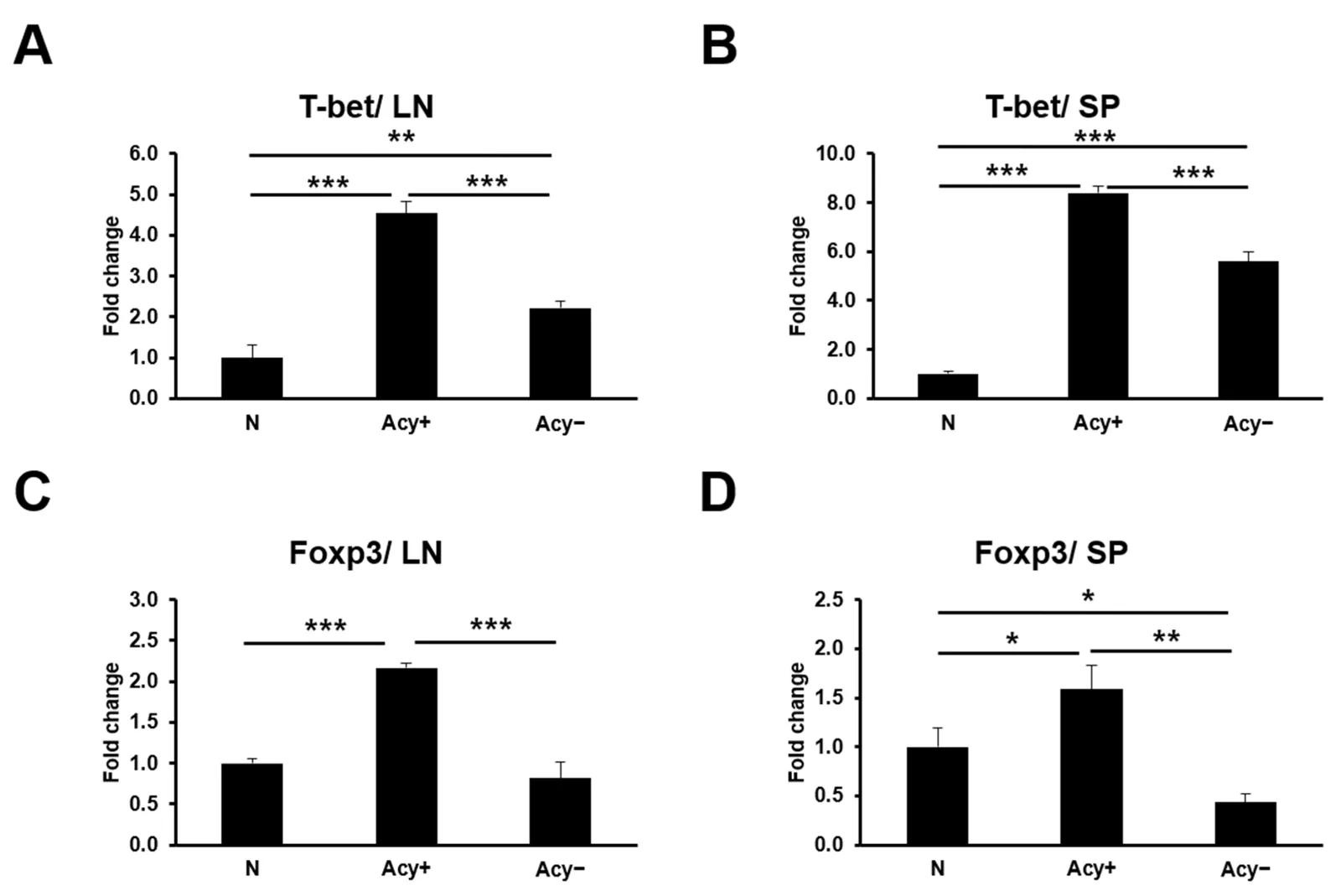
Effects of acyclovir on immune-related gene expression. (**A**–**D**) Expression of T-bet and Foxp3 in lymph node and spleen tissues was quantified by real-time RT-PCR. HSV-1 infection increased the expression of both genes compared with the normal control group. Treatment with acyclovir further enhanced the expression of T-bet and Foxp3 relative to untreated infected mice. Data are presented as the mean ± SEM. Statistical significance was determined as indicated (* *p* < 0.05, ** *p* < 0.01, *** *p* < 0.001).

HSV-1 infection increased the expression of T-bet and Foxp3 in both the spleen and lymph nodes compared with normal controls, consistent with activation of the host immune response. Notably, acyclovir treatment further increased the expression of both transcription factors relative to untreated infected mice (Figure 3A–D). These findings suggest that acyclovir may enhance immune regulation, potentially by modulating the balance between effector and regulatory T cell responses.

## 4. Discussion

The present study established a simple, practical, and reproducible murine model of localized cutaneous HSV-1 infection that recapitulates the characteristic skin lesions observed in human HSV-1 infection. The development of visible vesicular and ulcerative lesions is a hallmark clinical feature of HSV-1 infection and provides an important endpoint for investigating viral pathogenesis and therapeutic responses. However, many conventional murine models fail to consistently reproduce these cutaneous manifestations, often resulting in subclinical infection or rapid systemic dissemination without well-defined localized lesions. Therefore, a model that reliably generates localized skin lesions provides substantial value for translational studies of HSV-1 pathogenesis and antiviral therapeutics.

In this model, superficial scarification of the ear auricle followed by direct HSV-1 inoculation consistently induced localized skin lesions, indicating that disruption of the epithelial barrier is critical for efficient viral entry and lesion development. Intact skin functions as both a physical and an immunological barrier against infection, and superficial tissue injury likely facilitates viral access to epithelial cells and sensory nerve endings, thereby promoting local viral replication [19,20]. Productive HSV-1 infection depends on the coordinated activity of the viral DNA replication machinery, including the ribonucleotide reductase subunits encoded by UL39 and UL40, which are essential for deoxyribonucleotide synthesis and viral DNA replication [21,22]. Thus, successful lesion formation requires both effective viral entry and a permissive host environment that supports viral replication.

Administration of hydrocortisone further increased the incidence of lesion formation, suggesting that transient modulation of host immune responses facilitates disease development. This observation is consistent with previous evidence that temporary immunosuppression enhances viral replication and promotes lesion formation. Importantly, lesions remained confined to the facial region without evidence of systemic disease, supporting the suitability of this model for investigating localized cutaneous HSV-1 infection rather than disseminated infection.

Acyclovir remains the first-line antiviral therapy for HSV infections because of its selective inhibition of viral DNA synthesis [9,11]. In the present study, acyclovir treatment reduced viral gene expression and markedly decreased lesion size, demonstrating the utility of this model for evaluating antiviral efficacy. Although viral gene expression was not completely normalized, the observed partial suppression is consistent with the established mechanism of acyclovir as an inhibitor of viral DNA polymerase. These findings indicate that the model is sufficiently sensitive to detect therapeutic responses at both the molecular and clinical levels.

Interestingly, acyclovir treatment was associated with increased expression of both T-bet and Foxp3, suggesting concurrent activation of effector and regulatory immune pathways. T-bet is a key transcription factor that promotes Th1 differentiation and interferon-γ production [23], whereas Foxp3 is the master regulator of regulatory T cells [24]. However, the concurrent upregulation of these transcription factors should not be interpreted as evidence of a direct immunomodulatory effect of acyclovir. HSV-1 has evolved multiple mechanisms to evade host immunity, including inhibition of antigen presentation [25], suppression of interferon signaling [26], and modulation of adaptive immune responses [27]. Therefore, the increased expression of T-bet and Foxp3 following acyclovir treatment may reflect recovery of host immune function secondary to reduced viral replication and the alleviation of virus-mediated immune suppression, rather than a direct pharmacological effect of the drug [28].

Although acyclovir significantly reduced UL39 (R1) expression, UL40 (R2) expression showed only a modest reduction and remained relatively variable. The biological significance of this difference remains unclear and may reflect differential regulation of the viral ribonucleotide reductase subunits or biological variability associated with the limited sample size. Accordingly, this finding should be interpreted cautiously until confirmed by further mechanistic studies.

Several limitations of this study should be acknowledged. First, transcription factor expression was analyzed in whole tissue samples; therefore, the specific cellular sources responsible for the increased T-bet and Foxp3 expressions could not be identified. Future studies using flow cytometry or immunohistochemical analyses will be necessary to characterize the relevant immune cell populations and determine their functional significance. Second, direct measurements of viral load at the lesion site, including plaque assays or quantitative PCR for HSV-1 DNA, as well as histopathological evaluation of the lesions, were not performed. Therefore, HSV-1 ribonucleotide reductase gene expression was used as an indirect indicator of viral replication, and the extent of local tissue damage could not be comprehensively characterized. Finally, despite independent evaluation to minimize observer bias, the relatively small sample size remains limitations of the present study. Future studies incorporating larger cohorts, direct viral quantification, and more comprehensive immunological analyses will further validate and extend these findings.

Although additional studies are required to further characterize the underlying immune mechanisms and directly quantify viral burden, the present findings demonstrate that this model is capable of detecting both virological and clinical responses to antiviral treatment. The observed reductions in lesion severity and viral gene expression following acyclovir administration, together with the associated changes in T-bet and Foxp3 expression, suggest that suppression of viral replication may facilitate recovery of host immune responses. These findings suggest that this model provides a valuable experimental platform for investigating HSV-1 replication, host immune responses, and antiviral interventions. Incorporating direct viral quantification and histopathological analyses in future studies will further enhance the utility and translational relevance of this model.

## 5. Conclusions

This study establishes a simple and practical mouse model of localized cutaneous HSV-1 infection that consistently produces localized skin lesions under the experimental conditions used. Acyclovir treatment significantly reduced lesion size and viral gene expression, demonstrating that the model is capable of detecting antiviral efficacy. These findings suggest that the model may serve as a useful experimental platform for investigating HSV-1 pathogenesis and evaluating candidate antiviral therapies. Further studies incorporating direct viral quantification at the lesion, histopathological evaluation, and validation across independent laboratories will further establish the robustness, reproducibility, and broader applicability of this model.

## Figures and Tables

**Table 1:** Primer sequences used for qRT-PCR analysis.

Gene	Primer	Sequence (5′–3′)
β-actin	Forward	TGTCCACCTTCCAGCAGATGT
	Reverse	AGCTCAGTAACAGTCCGCCTAG
HSV-1 RR (UL39)	Forward	GGCTGCAATCGGCCCTGAAGTA
	Reverse	GGTGGTCGTAGAGGCGGTGGAA
HSV-1 RR (UL40)	Forward	CTTCCTCTTCGCTTTCCTGTCG
	Reverse	CGCTTCCAGCCAGTCCACCTT
T-bet	Forward	ATGTTTGTGGATGTGGTCTTGGT
	Reverse	CGGTTCCCTGGCATGCT
Foxp3	Forward	CACAATATGCGACCCCCTTTC
	Reverse	AACATGCGAGTAAACCAATGGTA

## Data Availability

Data is contained within the article.
